# Amyloid beta biomarker for dementia detection by hyperspectral ophthalmoscope images

**DOI:** 10.18632/aging.206171

**Published:** 2024-12-06

**Authors:** Yu-Bun Ng, Sheng-Feng Sung, Hong-Thai Nguyen, Shih-Wun Liang, Yu-Ming Tsao, Yi-Hui Kao, Wen-Shou Lin, Hsiang-Chen Wang

**Affiliations:** 1 Department of Radiology, Ditmanson Medical Foundation Chia-Yi Christian Hospital, Chiayi City 60002, Taiwan; 2 Department of Internal Medicine, Division of Neurology, Ditmanson Medical Foundation Chia-Yi Christian Hospital, Chiayi City 60002, Taiwan; 3 Department of Mechanical Engineering, National Chung Cheng University, Min Hsiung, Chia Yi 62102, Taiwan; 4 Department of Medical Education and Research, National Taiwan University Hospital Yun-Lin Branch, Douliu 640, Taiwan; 5 Department of Internal Medicine, Neurology Division, Kaohsiung Armed Forces General Hospital, Kaohsiung City 80284, Taiwan; 6 Director of Technology Development, Hitspectra Intelligent Technology Co., Ltd., Kaohsiung City 80661, Taiwan

**Keywords:** dementia detection, hyperspectral imaging, amyloid beta

## Abstract

The escalating prevalence and economic burden of dementia underscore the urgency for innovative detection methods. This study investigates the potential of hyperspectral imaging (HSI) to detect dementia by analyzing retinal changes associated with amyloid beta (Aβ) formations. Leveraging a dataset of 3,256 ophthalmoscopic images from 137 participants aged 60 to 85 years, categorized into dementia and non-dementia groups via the Mini-Mental State Examination (MMSE), we extracted features from five key regions of interest (ROIs) identified for their pronounced changes in Aβ biomarkers. The analysis revealed that gender does not significantly influence dementia levels, and no substantial spectral differences were observed within the 380 nm to 600 nm wavelength range. However, significant variations in spectral reflection intensity were noted between 600 nm and 780 nm across both genders, suggesting a potential avenue for distinguishing stages of dementia. Despite the impact of diabetes on the vascular system, its stages did not significantly influence dementia development. This research highlights the utility of HSI in identifying dementia-related retinal changes and calls for further exploration into its effectiveness as a diagnostic tool, potentially offering a non-invasive method for early detection of dementia.

## INTRODUCTION

The retina is a complex structure located in the inner layer of the eye wall. It is thin, with a thickness of approximately 250 μm, and is composed of ten histological layers ranging from the retinal pigment epithelium to the inner limiting membrane [1, 2]. Similar to the negative film of a camera, the retina is responsible for photoreception. A variety of eye diseases, including age-related macular degeneration [3], glaucoma [4, 5], diabetic retinopathy [6–8], retinitis pigmentosa [9], and macular edema [10], vitreous floater [11], may occur in the retina. Dementia encompasses many neurological disorders, including Alzheimer’s disease [12–14], vascular dementia [15], frontotemporal lobe degeneration [16], Parkinson’s disease dementia [17, 18], and Lewy body dementia [18, 19]. Dementia is a neurocognitive disorder characterized by memory deficits, cognitive decline, psychiatric and behavior problems. The World Health Organization listed this condition as the seventh leading cause of death globally in 2019 [20]. The International Association for Dementia (Alzheimer’s Disease International) reports that one person develops dementia every 3 s worldwide, with a current estimate of over 50 million individuals being affected [21, 22]. In 2018, the annual cost related to dementia was projected to exceed 1 trillion U.S. dollars globally [23]. In Taiwan, the Taiwan Alzheimer’s Disease Association reported that 18.01% of elderly individuals over 65 years old suffer from mild cognitive impairment (MCI), with 7.64% diagnosed with dementia. As a result, the total population of individuals with dementia reaches 312,166, accounting for 1.34% of the country’s population. The association also estimates that this number will reach 840,000 by 2070, equating to one dementia patient for every 20 people [24].

The alteration of amyloid beta (Aβ) serves as a biomarker for assessing the severity of dementia. The retina is a significant point of interest in understanding dementia for several reasons. While its primary role is in photoreception, serving as the eye’s light-sensitive layer, its connection to dementia stems from the fact that the retina is considered an extension of the central nervous system (CNS). Like the brain, the retina is affected by neurodegenerative processes, such as the accumulation of amyloid beta (Aβ), a key biomarker in Alzheimer’s disease. This makes the retina a valuable non-invasive window to detect early signs of dementia. Changes in the retina, such as Aβ deposits, can be detected through advanced imaging techniques like hyperspectral imaging, providing an accessible way to study CNS-related pathology without needing brain scans. Although the retina’s role in photoreception itself is not directly linked to dementia progression, its neural connection to the brain makes it useful for detecting dementia-related changes. Therefore, the retina serves as both a marker for neurodegeneration and a practical tool for early dementia detection. When subjected to light spectrums from hyperspectral imaging, Aβ exhibits a light scatter effect, which makes it a promising candidate for wavelength-based biomarker in dementia detection. Aβ is one of the main components that make deposits and plaques in the aging organs of Alzheimer’s disease (AD). Histological examination revealed that there is a relationship between increased Aβ plaques and neuronal loss in AD patients [25]. The process of Aβ fibril formation involves the generation of oligomers and subsequent progression towards the formation of “seeds,” which are considered the primary source of aging plaques [26, 27]. Multiple studies have indicated that the presence of Aβ leads to an increase in reflectance at shorter wavelengths (<550 nm) [28, 29]. However, it is important to note that this variation is not consistently observed across different investigations. The change in reflectance is influenced by factors such as the ophthalmoscope system used or alterations in the chemical composition of the solute Aβ. Notably, More and Vince [30] conducted a study using a darkfield microscope system, which yielded results contradictory to those observed by Hadoux et al. [28] and Lim et al. [29] when employing illumination solely from bright field mode. Hadoux et al. [28] reported an increase in reflectance at shorter wavelengths due to the presence of Aβ in patients with AD. Lim et al. [29] also acknowledged alterations in hyperspectral reflectivity at shorter wavelengths. Cheng et al. [26] utilized an LED-based interferometric reflectance sensor and employed an additional approach of incorporating active ingredients to either induce or inhibit the self-aggregation of Aβ. The findings of this study corroborated an elevation in reflectance at specific wavelengths when active substances promoting Aβ aggregation were present. Conversely, a decrease in reflectance was observed when Aβ inhibitors were introduced, demonstrating their impact on reflectance properties. Previous studies have explored the potential role of Aβ as a contributing factor, given its impact on light reflection and the correlation between Aβ concentration and this optical phenomenon. Additionally, these studies have identified Aβ as a biomarker for detecting dementia in clinical trials involving mice and similar experiments conducted on the human brain. The optical properties of Aβ have shown significant potential under HS signals. Many studies have focused on exploring the relationship between ophthalmoscope images and the burden of Aβ in the brain, particularly under the influence of hyperspectral signals. This area of research has garnered considerable attention due to its implications for understanding and detecting Aβ-related pathologies [31].

In this study, hyperspectral (HS) signals were derived from data at five locations: two located above the temporal vascular arch (S1 and S2), at the fovea (F), and two below the temporal vascular arch (I1 and I2), within ophthalmic images. These locations were selected due to the pronounced changes observed in Aβ biomarkers, facilitating the investigation of the relationship between dementia and various factors including personality traits, age, and underlying medical conditions such as diabetic retinopathy. The dataset comprised 3,256 ophthalmoscopy images from 137 participants, aged between 60 and 85 years, who were categorized into two groups—those with and without dementia—based on the Mini-Mental State Examination (MMSE) for classification. Additional participant information collected included age, gender, and diabetic retinopathy status. Features from five regions of interest (ROIs) were extracted using hyperspectral imaging technology, with ROIs being determined by ophthalmologists according to standardized guidelines. The methodological approach involved the application of information analysis techniques. The findings indicate that gender does not significantly influence dementia levels, as evidenced by a chi-square statistic of 0.778, a *p*-value of 0.678, and a Cramer’s V of 0.075, all suggesting a weak association. Furthermore, spectral analysis across different stages of dementia revealed no significant gender differences or stage-specific differences within the 380 nm to 600 nm wavelength range. However, notable differences in spectral reflection intensity were observed between 600 nm and 780 nm, consistently across both genders. This research underscores significant variations in reflectance spectra at longer wavelengths due to Aβ formations and aging, highlighting their potential utility in detecting stages of dementia, though further investigation is required to clarify these associations. Despite the impact of diabetes on the eye’s vascular system, the study concludes that the stages of diabetes do not significantly influence dementia development, as supported by statistical analyses of diabetic ophthalmoscopy images and patient samples.

## MATERIALS AND METHODS

### Data collection

To accurately distinguish the types of dementia, we employed the MMSE as the evaluation criterion in this study. The MMSE, proposed by Folstein et al. [32] and further developed for the early detection of dementia in people with MCI [33, 34], consists of five major components, including orientation to time and place, short-term memory, attention and calculation abilities, language abilities, and visual-spatial abilities, with a total score in the range of 0–30 points. As shown in Table 1, 0–22 points indicate dementia, 23–27 points suggest MCI, and 28–30 points indicate normal cognitive function. The MMSE [32] is a simple and reliable test and is widely used to measure the severity and progression of cognitive impairment. It is a 30-point questionnaire and examines functions including orientation, registration, attention and calculation, recall, language, repetition and complex commands. Although the MMSE does not provide a diagnosis of any specific disease [35], an MMSE score of 22 or less has been reported to distinguish well between DSM-III-R/ICD-10 dementia and mild cognitive impairment (92% specificity and 96% sensitivity) [33]. MMSE can provide a preliminary evaluation and help avoid the unnecessary utilization of medical resources. The process of collecting data from patients is often beyond the control of researchers and influenced by external factors such as the number of patients available for examination during the study period and ethical considerations. In our study, the distribution of participants across the three disease classes was relatively balanced, with 49 participants classified as Normal, 54 patients as MCI, and 34 patients as Dementia. The number of images for each category was 1254 for normal, 1320 for MCI, and 682 for dementia. Additionally, the sex ratio of patients was not significantly imbalanced, with a slightly higher number of female patients than male patients. The study participants’ ages ranged from 60 to over 80 years old, which is the age range most likely to experience dementia symptoms. Accompanying data for each photograph, collected via ophthalmoscopy, include age, gender, and underlying medical conditions, specifically categorized according to the stage of diabetic retinopathy. To ensure confidentiality and data protection, participants’ personal information and any other data not pertinent to this study were encrypted. The statistics among dementia patients is shown in Table 2.

**Table 1:** MMSE, short intelligence test scores, and classification into three categories (normal, MCI, and dementia).

**Class**	**MMSE**
Normal	28–30
MCI	23–27
Dementia	0–22

**Table 2:** Detailed statistics by age group, sex, and MMSE score among dementia patient groups.

	**Dementia**	**MCI**	**Normal**	***p*-value**	**95% CI**
No. of patients (Male/Female)^1^	24/34	18/22	16/23	0.9206	
Age^2^	74.98 ± 6.15	76.60 ± 5.93	76.13 ± 6.33	0.4056	Dementia vs. MCI: (−4.02, 0.78)
					Dementia vs. Normal: (−3.66, 1.37)
					MCI vs. Normal: (−2.20, 3.14)
MMSE^2^	17.47 ± 4.46	25.38 ± 1.58	29.15 ± 0.78	<0.0001	Dementia vs. MCI: (−9.15, −6.67)
					Dementia vs. Normal: (−12.85, −10.53)
					MCI vs. Normal: (−4.32, −3.24)
No. of images	682	1320	1254		
DR (Normal/BDR/PDR/PPDR)	11/10/18/19	6/10/9/15	11/6/11/11	0.006	

Abbreviations: DR: diabetic retinopathy; BDR: background retinopathy; PDR: preproliferative retinopathy; PPDR: proliferative retinopathy. ^1^Categorial variables are expressed as number of participants. A chi-squared test to compare the observed frequencies of males and females across the groups. ^2^Interval variables are expressed as mean ± standard deviation. ANOVA (Analysis of Variance) to compare the mean age and MMSE score across the three groups. The 95% CI is the difference between means.

### Data processing

**Figure 1:**
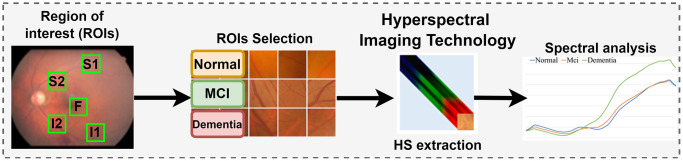
**Experimental flow chart.** Hyperspectral imaging technology was utilized to extract spectral features from five designated positions, known as Regions of Interest (ROIs), within the augmented dataset. This dataset was developed through a manual process based on the MMSE. Spectral analysis was then applied to these ROIs. The diagram highlights the cropped region of the fundus image as the Region of Interest (ROI), with the sampling positions located above the temporal vascular arcade (S1 and S2), at the fovea (F), and below the temporal vascular arcade (I1 and I2).

Figure 1 illustrates the comprehensive research process conducted in this study. The research employed hyperspectral imaging technology to capture the spectrum of a specific area within the image and analyze the spectral differences between lesions. Additionally, hyperspectral imaging technology was utilized to extract features from five ROIs within an augmented dataset. Spectral analysis was then applied to the ROIs to facilitate preliminary classification based on the MMSE. The collected ophthalmoscopic images were sampled in specific areas. The regions are selected to ensure correlation between spectral variables and to avoid selection bias. Sampling areas with high color contrast representation because of the presence of vascular organizations, and biological organs of the eye such as fovea and nerve fiber, help to enhance spectra bands [28]. The sampling locations were set in five positions: above the temporal vascular arcade (S1 and S2), the fovea (F), and below the temporal vascular arcade (I1 and I2). The area size was 240 × 240 pixels. The images were then transformed into 401 bands of visible-light spectrum information using hyperspectral conversion technology. HS imaging algorithm for ophthalmoscopic images is described in our previous studies [6, 36]. The resulting spectral information contained 401 spectral channels within the visible-light band (380 nm to 780 nm). The five positions, S1, S2, F, I1, and I2, are obtained as hyperspectral signals. The spectral intensity is calculated as the mean value in each region (the mean value following the spatial dimension 240 × 240) and then represented on the corresponding diagrams. The stages of dementia, including normal, MCI, and dementia, are detected by analyzing the differences in spectral intensity on the diagram.

### Hyperspectral fundus imaging

#### Hyperspectral fundus camera system

**Figure 2:**
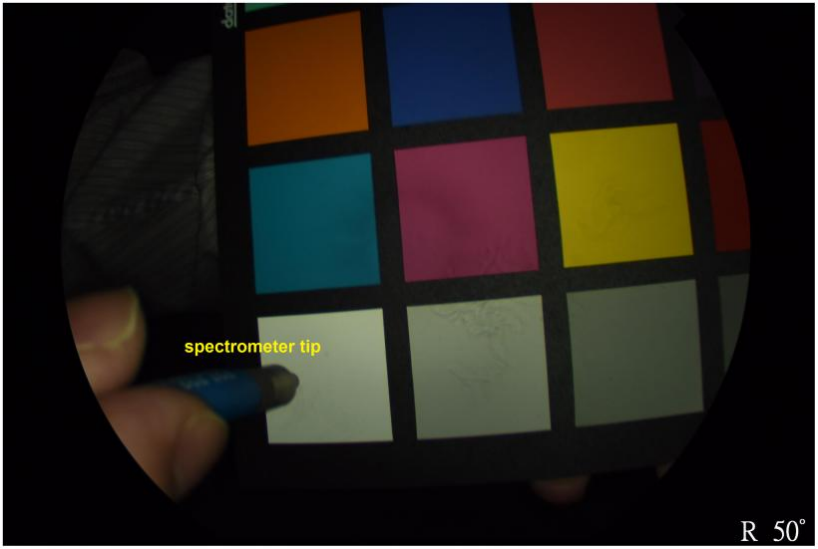
The spectra were collected utilizing a 24-color checker reference and a spectrometer (Ocean Optics, QE65000), with simultaneous measurements of the light source spectrum.

The hyperspectral system used in this study consists of a TRC NW8/8F fundus system combined with a DSLR camera, supporting Color, Red-Free, and Fluorescein Angiography imaging. It features a 16.2-megapixel camera, providing high-resolution images with a 45° field of view. The system includes nine internal fixation points, enabling the capture of wide-angle retinal views. Spectral data were collected using a spectrometer (Ocean Optics, QE65000) from a 24-color reference chart, specifically a 24-color checker. Additionally, the spectrum of the light source used in the fundus camera was recorded (Figure 2).

#### Hyperspectral fundus camera system

**Figure 3:**
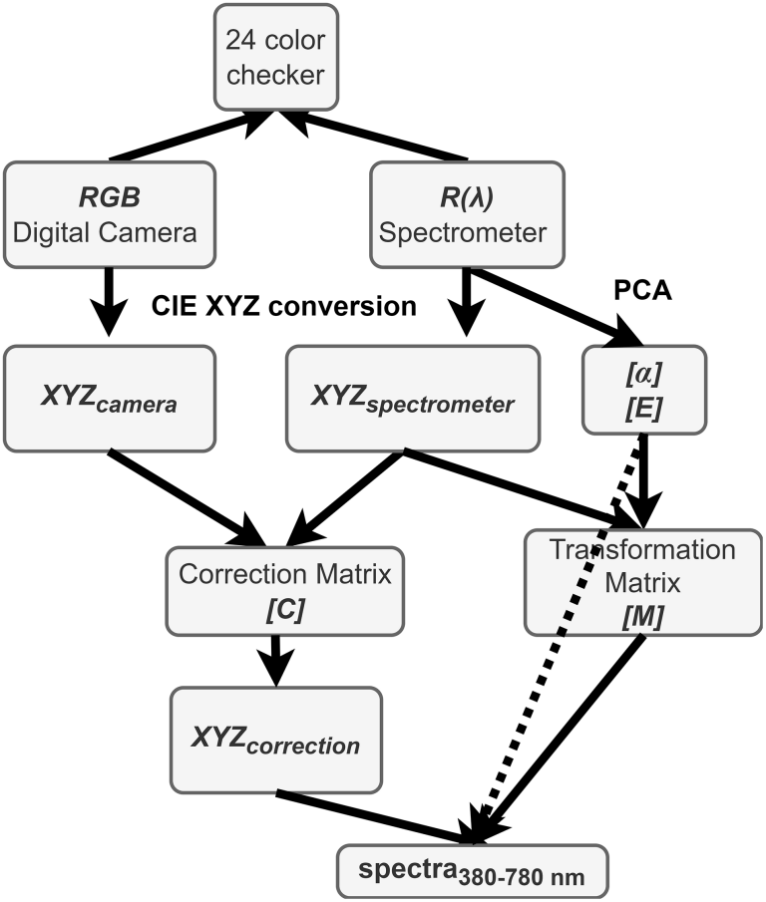
Schematic diagram of the proposed method.

Hyperspectral fundus imaging algorithm was employed using an ophthalmoscope (Kowa Nonmyd 7) and a spectrometer (Ocean Optics, QE65000) to establish the correlation between the two devices by capturing a shared target. The standard 24-color card (X-Rite Classic, 24 Color Checkers) served as the common target. The calibration between fundus camera and spectrometer is performed by correcting the signal received from those devices through the 24-color chart. The resultant image was then converted into a set of sRGB channel values. From these datasets, a transformation matrix was derived. The entire modeling process is depicted in Figure 3.

Under identical lighting conditions, a digital camera is utilized to capture images of color checkers. The captured images are saved in the sRGB format, specifically as JPEG files. This process involves normalizing the values of the three primary color channels—Red (R), Green (G), and Blue (B)—from their original range of approximately 0–255 to a normalized range of approximately 0–1. Subsequently, the CIE XYZ tristimulus values are derived by converting these normalized RGB values into *XYZ* values as *XYZ_camera_*. This conversion is facilitated through the application of a specific transformation formula, detailed below:


$$\left[\begin{array}{c} X\\ Y\\ Z \end{array}\right]=[T]\left[\begin{array}{c} f(R_{sRGB})\\ f(G_{sRGB})\\ f(B_{sRGB}) \end{array}\right]\times 100,0\leq \begin{array}{c} R_{sRGB}\\ G_{sRGB}\\ B_{sRGB} \end{array}\leq 1\;\;(1)$$

where 0≤ *R_sRGB_*, *G_sRGB_*, *B_sRGB_* ≤1. Here, *R_sRGB_*, *G_sRGB_*, and *B_sRGB_* denote the three color channels of the sRGB image. These channel values are linearized through a function defined by gamma correction. For sRGB images, this correction can be approximated by a power function with an exponent of 2.2, incorporating a linear segment for lower values to account for the non-linear sensitivity of human vision at these intensities. The formula for removing gamma correction, or linearization, is defined as follows:


$$f(n)=\{\begin{array}{c} {\left(\frac{n+0.055}{1.055}\right)}^{2.4},\;\;n\;\;>0.04045\\ \left(\frac{n}{12.92}\right),\;\;otherwise \end{array} \;\;(2)$$

and the transformation matrix T is defined by:


$$\left[T]=\left[\begin{array}{ccc} 0.4104 & 0.3576 & 0.1805\\ 0.2126 & 0.7152 & 0.0722\\ 0.0193 & 0.1192 & 0.9505 \end{array}\right]\;\;(3\right)$$

Converting the obtained spectral data *R(λ)* (380 nm ~ 780 nm, 1 nm) into the XYZ color gamut space requires the light source spectrum *S(λ)* of the ophthalmoscope hyperspectral system, and the XYZ color matching function. Through equations 4, 5, and 6, the spectral data are converted into the XYZ values as *XYZ_spectrometer_*.


$$X=k\int_{380\;nm}^{780\;nm}S(\lambda )R(\lambda )\bar{x}(\lambda )\;\;d\lambda \;\;(4)$$

$$Y=k\int_{380\;nm}^{780\;nm}S(\lambda )R(\lambda )\bar{y}(\lambda )\;\;d\lambda \;\;(5)$$

$$Z=k\int_{380\;nm}^{780\;nm}S(\lambda )R(\lambda )\bar{z}(\lambda )\;\;d\lambda \;\;(6)$$

The brightness ratio *k*, as shown in Equation 7:


$$k=100/\int_{380\;nm}^{780\;nm}S(\lambda )\bar{y}(\lambda )\;\;d\lambda \;\;(7)$$

where *S(λ) is* the light source spectrum in XYZ color gamut space, $\bar{x}(\lambda )$, $\bar{y}(\lambda )$, and $\bar{z}(\lambda )$ are the *XYZ* values of color matching function. The spectral data signal *R(λ)* from the spectrometer are converted to the XYZ color space.

The color correlation between the spectrophotometer and camera was obtained; the regression matrix (C) is defined as follows:


$$\left[C]=\left[XYZ_{spectrometer}\right]\times pinv([V])\;\;(8\right)$$

where *V* is defined as:


$$\begin{array}{c} V=[X^{3}\;Y^{3}\;Z^{3}\;X^{2}Y\;X^{2}Z\;Y^{2}\;Z\;XY^{2}\;XZ^{2}\;YZ^{2}\\ XYZ\;X^{2}\;Y^{2}\;Z^{2}\;XY\;XZ\;YZ\;X\;Y\;Z\;1{]}^{T} \end{array}\;\;(9)$$

with *X, Y*, and *Z* components derived from *XYZ_camera_*, and (*XYZ_spectrometer_*) is matrix constituted by *X, Y*, and *Z* components obtained from the spectrometer.

Equation 9 is also employed to extend the (*XYZ_camera_*) as the (*XYZ_camera_extend_*) resulting in the corrected values between the camera and spectrometer, denoted as *XYZ_correction_*, as delineated in Equation 10:


$$\left[XYZ_{correction}\right]=\left[C\right]\times \left[XYZ_{camera\_extend}\right]\;\;(10)$$

where (*XYZ_camera_extend_*) is defined as Equation 9 with *X, Y*, and *Z* components derived from *XYZ_camera_*, and (*C*) is correction matrix obtained from Equation 8.

The spectra were organized into a matrix, denoted as (*R(λ)*)_401 × 24_, where the columns represent the number of color checkers, and the rows correspond to the intensities of the wavelengths at 1 nm intervals. 12 eigenvectors, identified as having the most significant contribution, were established as the basis for spectral calculation and organized into a matrix (*E*)_12 × 401_ by calculating the eigen system and utilizing principal component analysis (PCA). The corresponding eigenvalues of these six eigenvectors, denoted as (*α*)_12 × 24_, were determined as follows:


$${\left[\alpha \right]}^{T}={\left[R(\lambda )\right]}^{T}pinv[E]\;\;(11)$$

Eventually, a transformation matrix (*M*) among the camera and spectrophotometer was determined by the following equation:


$$\left[M]=[\alpha ]pinv[V_{colour}]\;\;(12\right)$$

where *V_color_* is defined as Equation 13:


$$V_{color}={\left[XYZ\;XY\;YZ\;XZ\;X\;Y\;Z\right]}^{T}\;\;(13)$$

with *X, Y*, and *Z* components derived from *XYZ_spectrometer_*; (*α*) represents principal component scores obtained from 12 sets of principal components for dimensionality reduction of *R(λ)*.

The calculated spectra in the visible light range (380–780 nm) were determined according to the following equation:


$${\left[spectra\right]}_{380-780\;nm}=[E][M][XYZ_{camera}]\;\;(14)$$

### Data availability statement

The datasets used and/or analyzed during the current study are available from the corresponding author on reasonable request.

## RESULTS AND DISCUSSION

### Spectral analysis on biomarker locations

**Figure 4:**
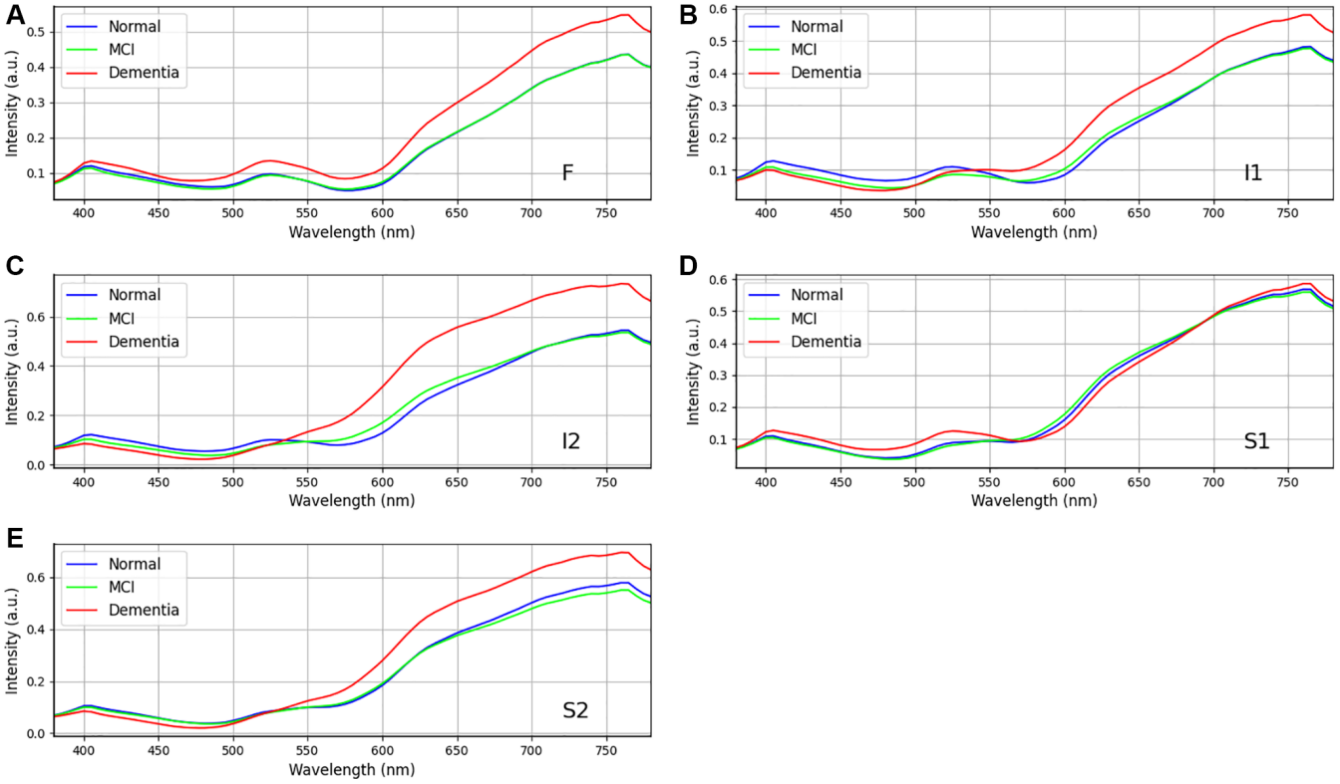
**Retinal spectra of the same age women in different degrees of dementia among dementia group (*n* = 24), MCI group (*n* = 18), and Normal group (*n* = 16).** (**A**–**E**) show the different region in the retina (**A**) F, (**B**) I1, (**C**) I2, (**D**) S1, and (**E**) S2. Specifically, positions F, I1, I2 and S2 show the difference in the long wavelength region (>550 nm). Data shown as mean normalized spectrum.

**Figure 5:**
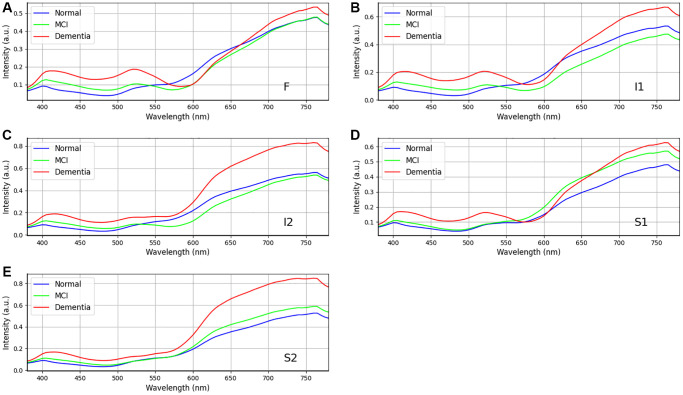
**Retinal spectra of the same age men in different degrees of dementia among dementia group (*n* = 34), MCI group (*n* = 22), and Normal group (*n* = 23).** (**A**–**E**) show the different region in the retina (**A**) F, (**B**) I1, (**C**) I2, (**D**) S1, and (**E**) S2. Positions I2 and S2 show the difference in the wavelength range from 600 to 750 nm. Data shown as mean normalized spectrum.

Statistical analysis was performed to examine the influence of gender on different levels of dementia on dataset in Table 2. The chi-square statistic is a measure of the overall association between gender and class in the observed contingency table. In this case, the chi-square statistic is 0.778, indicating a relatively low value. A larger chi-square statistic suggests a stronger association between the variables. The *p*-value associated with the chi-square statistic measures the probability of obtaining the observed association (or more extreme) between gender and class by chance alone, assuming that there is no true association in the population. In this case, the *p*-value is 0.678, which is greater than the typical significance level of 0.05. There is no significant difference in the distribution of genders among the three classes. Cramer’s V is an effect size measure for the chi-square test. It quantifies the strength of the association between gender and class, ranging from 0 to 1. A value closer to 0 indicates a weaker association, while a value closer to 1 indicates a stronger association. In this case, Cramer’s V is 0.075, suggesting a weak association between gender and class. Figures 4 and 5 show the retinal spectra of patients with different degrees of dementia for women and men of the same age, respectively. The results revealed that, in the area S1, no discernible spectral difference was observed. Furthermore, the variations among the three stages in the regions of F, I1, I2, and S2 were also found to be statistically insignificant within the wavelength range of 380 nm to 600 nm. However, when the wavelength was between 600 nm to 780 nm, the spectral reflection intensity of dementia was significantly higher than that of MCI and normal categories. This trend was consistent for women and men. The spectral reflection intensity of dementia was higher than those of MCI and normal conditions at the wavelengths of 380 nm to 600 nm in all five regions. However, the spectral reflection intensity of normal condition was higher than those of dementia and MCI at wave-lengths of 600 nm to 780 nm.

### Relationship of Aβ and aging and effects of aging Aβ on dementia

In our study, we observed a disparity in reflectance spectra primarily in the longer wavelength range (>550 nm), as depicted in Figures 4 and 5. As the result, it is possible to detect different stages of dementia on male and female. Our study’s findings suggest that while gender does not significantly influence dementia levels, there are observed differences in spectral reflection intensity between genders. These findings imply that while the prevalence or severity of dementia may not differ substantially between men and women, the way dementia-related changes, such as Aβ accumulation, manifest in the retina could vary by gender. The differences in spectral reflection intensity may reflect underlying biological or structural differences in the male and female retina, such as hormonal influences, vascular differences, or other factors that affect how light is absorbed or scattered in retinal tissues. These variations could affect how hyperspectral imaging detects dementia-related changes and suggest the need for gender-specific calibration in diagnostic imaging techniques. Additionally, while gender may not directly impact dementia risk or severity, these spectral differences could still have implications for understanding how dementia progresses differently in men and women at a physiological level. Future research could explore these findings to improve the accuracy of retinal imaging in dementia diagnosis across genders. Other factors, such as age, gender, and eye-related diseases, may affect the performance of the spectrum. Therefore, more research is required to establish a reliable foundation for spectral analysis of the influence of such factors on dementia. This discrepancy can be attributed to the distinct morphologies adopted by Aβ formations under varying conditions. The process of Aβ formation exerts an influence on the growth of fibrils, thereby contributing to the observed variations in reflectance.

In the realm of geriatrics, the predominate determinant of dementia is the progressive process of aging. Consequently, as individuals advance in years, they are confronted with a significantly escalated probability of manifesting symptoms of dementia. Statistical data indicate that in the age demographic of 65 to 69 years, approximately 2% of individuals are afflicted with this condition. Notably, the risk trajectory accentuates with advancing age, experiencing a near doubling in prevalence approximately every quintennial period. This translates to a substantial 33% prevalence rate amongst individuals who have surpassed the 90-year milestone. The correlation between aging and dementia can be attributed to the chronic progression of the disease, which is often instigated by underlying neurological disorders such as Alzheimer’s or vascular diseases. These ailments possess the propensity to inflict prolonged damage to the brain, ultimately culminating in the manifestation of dementia symptoms. Essentially, the extended lifespan augments the timeframe for the potential development of dementia, as the brain is exposed to a prolonged period of potential degenerative influences. Furthermore, aging serves as a facilitator for the onset of dementia due to the concurrent physiological and health alterations occurring during this period. These transformations, coupled with an inherent increase in physical frailty, can potentially exacerbate the risk of cognitive degradation, encompassing impairments in memory and critical thinking faculties. It is imperative to acknowledge that while the elderly population represents the highest risk group, dementia is not confined to this demographic. In a study conducted on 5xFAD mice, it was found that Aβ plaques increase with age. These findings suggest that light scatter is induced by the presence of oligomers in younger individuals. However, as we age, the oligomers diminish while Aβ plaques become more prevalent, potentially leading to enhanced scatter and a more pronounced effect at longer wavelengths. Our study reinforces the strong association between amyloid beta occurrence and light scattering, thereby contributing to the observed differences at longer wavelengths. This also aligns perfectly with the results from Hadoux et al. [28] when applying the spectral model to the validation dataset at sampling position S1, which is regarded as having the largest spectral difference. Nevertheless, an important aspect of our study was the influence of the retinal vascular system, specifically the arteries and veins. With age, the concentration of lutein in the retina decreases, resulting in shrinkage of the arteries and veins [11, 37–40]. This decrease in lutein concentration leads to a reduction in reflection intensity within the wavelength range of 580 nm to 780 nm, thus resulting in a significant difference in spectral intensity observed across various age groups.

**Figure 6:**
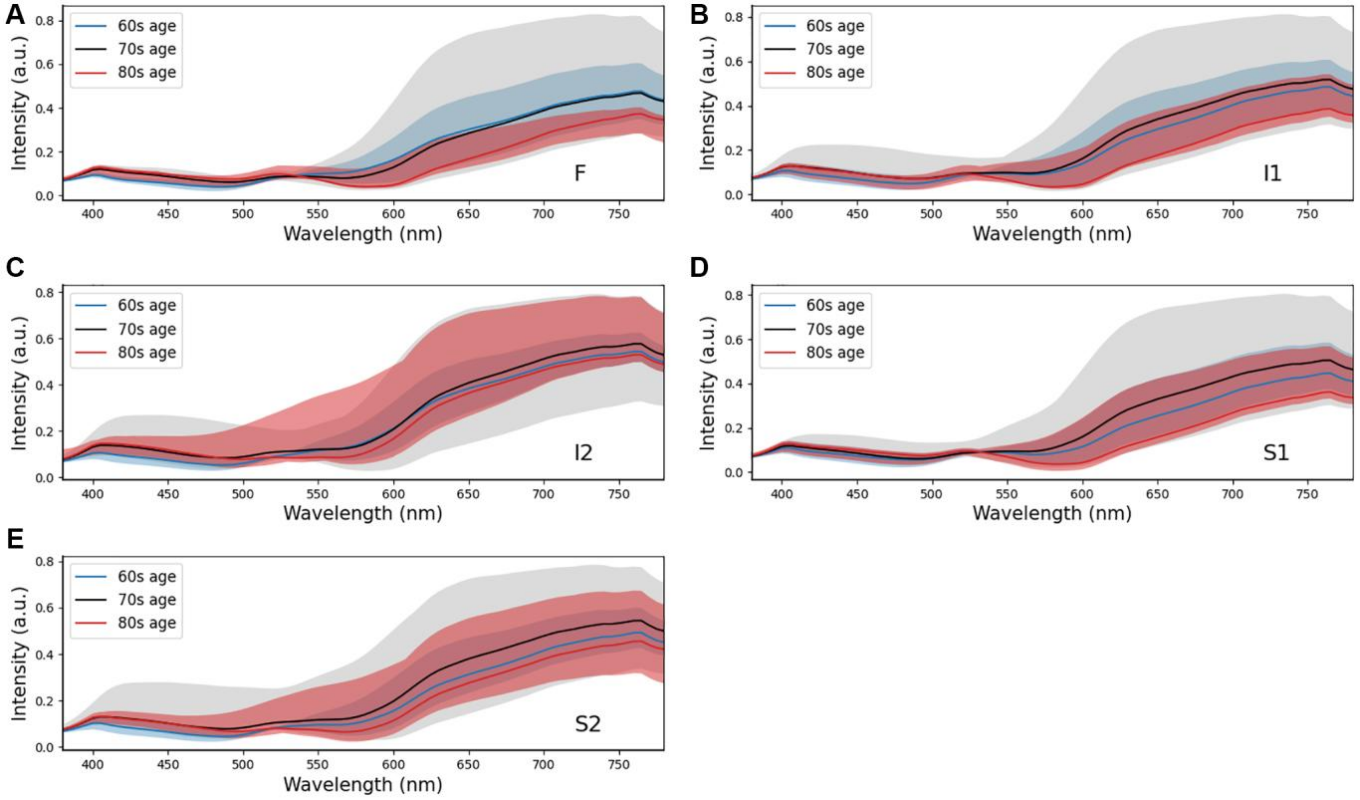
Comparison of retinal spectra by three age intervals (n_patients_ = 137, *p*-value = 0.032, Chi-square test and n_samples_ = 3,000, *p*-value < 0.006, Chi-square test) at sampling locations (**A**) F, (**B**) I1, (**C**) I2, (**D**) S1, and (**E**) S2. Positions F, I1, and S1 show the difference at long wavelengths (>550 nm).

The observed variations in longer wavelengths among the three different age groups can be attributed to the differential presence of Aβ plaques and oligomers. The profiles shown in Figure 6 demonstrate an increase in the difference of reflectance at longer wavelengths at five surveyed locations. This rough surface profile contributes to a scatter phenomenon when observed using hyperspectral imaging. The most pronounced difference is observed at the foveal location (F), as well as the superior (S1) and inferior (I1) locations. In our study, we selected three patients from three different age groups (60s, 70s, and 80s). The Chi-square statistical analysis was employed to assess a cohort comprising 137 patients and to scrutinize the spectra derived from 3,000 corresponding ophthalmoscope images. The resulting *p*-value, at 0.032, attests to statistical significance, thereby indicating that age may be considered a contributory factor in the etiology of dementia.

### The relationship between diabetes and dementia is independent

**Figure 7:**
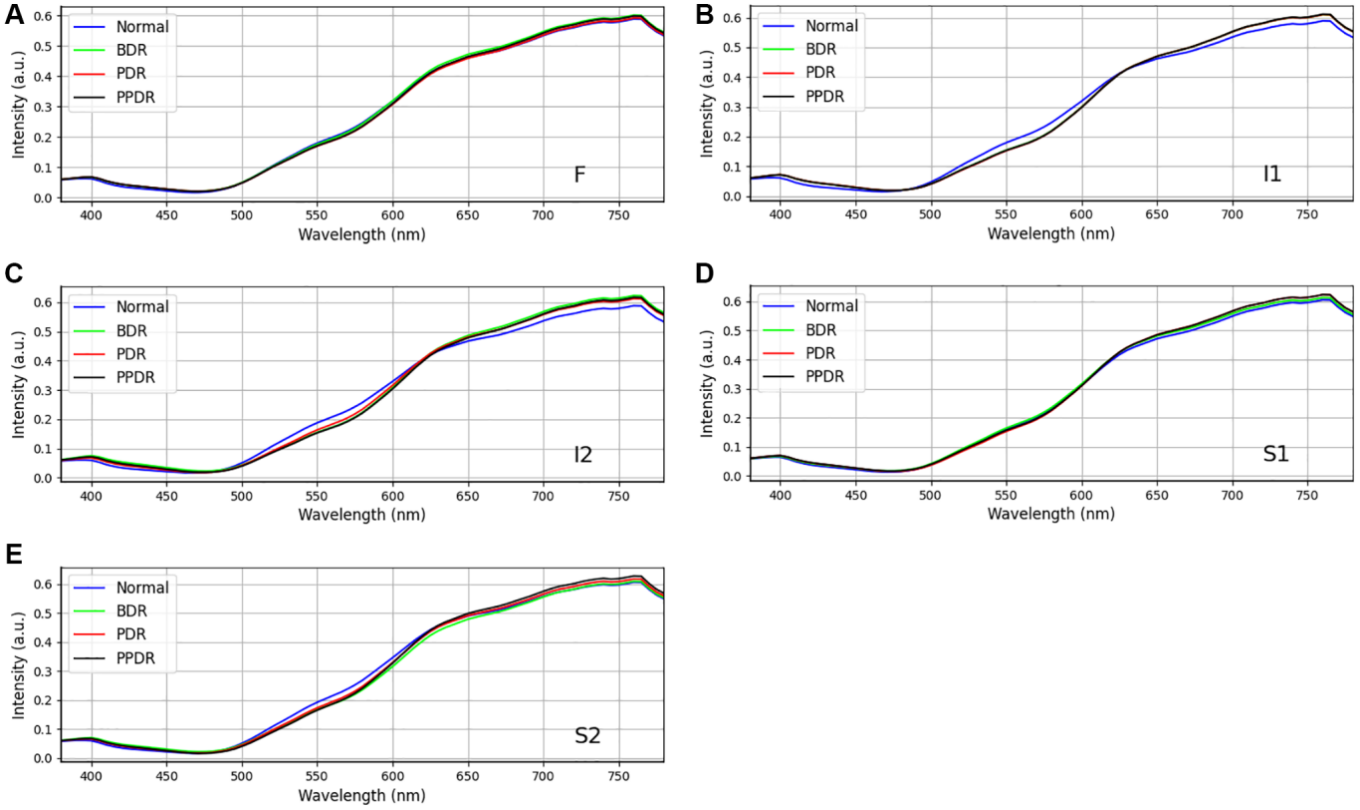
Comparison of retinal spectra by four stages of diabetes disease (n_patients_ = 137, p-value = 0.006, *Chi-square* test and n_samples_ = 3,000, *p*-value < 0.05, *Chi-square* test) at sampling locations (**A**) F, (**B**) I1, (**C**) I2, (**D**) S1, and (**E**) S2.

The relationship between dementia and diabetes has received limited attention in previous research. An investigation into the progression of diabetic retinopathy (DR) from mild to moderate and severe stages has proposed a potential link between diabetes and the development of dementia. However, it is crucial to acknowledge that these conclusions stem from clinical trials with a relatively small sample size. In an effort to mitigate potential analytical biases associated with diabetes, this study conducted an additional analysis of a spectrum of diabetic ophthalmoscopy images. The analysis primarily focused on assessing the oxygen concentration in the blood vessels of the retina in patients with diabetes as the basis for staging. The diabetic ophthalmoscopy image spectrum was categorized into four stages: *Normal*, background retinopathy (*BDR*), preproliferative retinopathy (*PDR*), and proliferative retinopathy (*PPDR*). In Figure 7, it is evident that the spectral regions show no substantial differences across the four stages of diabetes. This observation suggests that the various stages of diabetes predominantly impact the vascular system, encompassing both the arteries and veins within the eye. Conversely, the structural abnormalities associated with dementia are relatively minor in scale and are typically confined to the retina, often presenting with minimal external manifestations. The *p*-value obtained from the Chi-square statistical analysis of 137 samples from Table 2 (*p*-value = 0.006) and more than 3,000 ophthalmoscope images (*p*-value < 0.05) suggests sufficient statistical significance to support the conclusion that there are no significant influences of diabetes on dementia within the scope of this study.

## CONCLUSIONS

In the context of an aging population, the intersection of dementia and retinal diseases, traditionally associated with advanced age, is becoming more prominent as their onset age decreases. This shift underscores the urgency for ongoing innovation in drug development and medical diagnostics to enhance patient care, especially in early detection and treatment. Retinal imaging, including the use of HSI, stands out for its potential in diagnosing and treating conditions such as glaucoma and diabetic retinopathy. However, our study reveals that conventional RGB imaging may not offer significant advantages over HSI, indicating the necessity for further advancements in integrating HSI into medical imaging for improved predictive outcomes.

Our investigation employed HSI to analyze retinal images from 137 participants, aged 60–85, divided into groups with and without dementia, based on the MMSE. Spectral features were extracted from five specific ROIs identified for their relevance to changes associated with dementia biomarkers. Despite incorporating a comprehensive dataset, the study found no significant gender influence on dementia levels (chi-square = 0.778, *p*-value = 0.678, Cramer’s V = 0.075). Additionally, spectral analysis did not reveal significant differences across genders or dementia stages within the 380–600 nm wavelength range. However, the 600–780 nm range showed notable differences in spectral reflection intensity across all participants, suggesting the potential of this wavelength range in identifying dementia stages through Aβ formations and aging effects. Interestingly, our analysis indicated that diabetes stages do not significantly impact dementia development, challenging assumptions about the relationship between these two conditions. Furthermore, our study highlights the importance of further research to understand the spectrum’s performance across different factors like age, gender, and eye-related diseases, and how Aβ formation morphology affects spectral reflectance, especially in the longer wavelength range (>550 nm).

This study contributes to a nuanced understanding of the potential links between diabetes and dementia, suggesting minimal direct impact of diabetes stages on dementia development within our sample. It also emphasizes the need for larger-scale clinical trials and a broader analysis of diabetic ophthalmoscopy images to refine our understanding of these relationships and the role of retinal imaging in detecting and managing dementia and related retinal diseases effectively. The discussion emphasizes the significant role of aging in the development of dementia, particularly through the accumulation of Aβ in the retina and its impact on light scattering. As individuals age, Aβ accumulation increases, influencing reflectance at longer wavelengths, which is a key marker in dementia detection. However, aging also affects other retinal characteristics, such as vascular changes, a decrease in lutein concentration, and general tissue degeneration. These changes can act as confounding factors in detecting Aβ and interpreting retinal imaging results, making it difficult to isolate the effects of aging from those of dementia-related pathologies.

Future research will need to address these confounding factors to improve the accuracy of dementia detection in older populations. One way to do this is by refining imaging techniques to distinguish between Aβ-related changes and other aging-related retinal alterations. This could involve developing more sophisticated algorithms for hyperspectral imaging or combining retinal imaging with other diagnostic tools, such as brain imaging or blood-based biomarkers. By using multiple modalities, researchers could more accurately differentiate between normal aging processes and dementia-specific changes in the retina.

Additionally, longitudinal studies will be critical in understanding how aging interacts with Aβ accumulation and other retinal changes over time. These studies could track individuals from middle age through old age, monitoring how Aβ deposits progress and how other aging-related factors, like vascular health or macular degeneration, influence dementia risk. Understanding these interactions over time would allow researchers to pinpoint when and how Aβ accumulation becomes a significant contributor to dementia.

Lastly, future research should investigate how systemic factors, such as metabolic health, lifestyle, and genetics, influence both aging and retinal characteristics. These factors may accelerate or mitigate the effects of aging on the retina and could interact with Aβ deposition in complex ways. By taking a more holistic view of aging and retinal health, future studies can better control for these confounding influences and offer more precise insights into the relationship between aging, Aβ, and dementia development.
